# Perinatal depression and its impact on infant outcomes and maternal-nurse SMS communication in a cohort of Kenyan women

**DOI:** 10.1186/s12884-022-05039-6

**Published:** 2022-09-22

**Authors:** Alyssa D. Hummel, Keshet Ronen, Amritha Bhat, Brenda Wandika, Esther M. Choo, Lusi Osborn, Maneesh Batra, John Kinuthia, Manasi Kumar, Jennifer A. Unger

**Affiliations:** 1 University of Washington, Seattle, USA; 2 Kenyatta National Hospital, Nairobi, Kenya; 3 University of Nairobi, Nairobi, Kenya; 4 Department of Obstetrics and Gynecology, Warren Alpert Medical School Brown University, Women and Infants Hospital, Providence, USA; 5 Department of Global Health, University of Washington, Seattle, USA

**Keywords:** Perinatal depression, mHealth intervention, SMS messaging, Kenya

## Abstract

**Background:**

Perinatal depression is broadly defined as depressive symptoms during pregnancy or within the 12 months following delivery, affecting approximately 20–25% of pregnant and postpartum women in low- and middle-income countries. The wide accessibility of mobile phones allows mobile health (mHealth) interventions to be considered a solution to identify perinatal depression and provide appropriate referrals for treatment. This study, nested in a larger SMS communication project, examined the prevalence and correlates of perinatal depression, determined the association between antenatal depression and infant morbidity and mortality, and compared SMS communication patterns between women with and without perinatal depression.

**Methods:**

This was a prospective longitudinal cohort study of pregnant women seeking antenatal services at two public sector health clinics in Kenya. SMS messages were sent to participants with educational content related to their pregnancy and infant health and two-way SMS communication occurred with a nurse. Sociodemographic and obstetric characteristics, SMS messaging behaviors, infant health status, and depressive symptoms were assessed by a standardized questionnaire administered at enrollment (30–36 weeks gestation) and follow-up (14 weeks postpartum). Generalized estimating equation (GEE) with Poisson link was used to evaluate correlates of perinatal depressive symptoms, infant outcomes, and frequency of SMS messaging.

**Results:**

Of the 572 women with complete follow-up information, 188 (32.9%) screened positive for elevated depressive symptoms (≥10 by EPDS scale) at some time point during pregnancy or postpartum. The strongest predictors of any depressive symptoms included interpersonal abuse during pregnancy, fewer years of schooling, and maternal unemployment. Antenatal depressive symptoms were associated with an increased risk of infant illness or hospitalization (RR = 1.12, 95% CI: 1.11, 1.13). Women with antenatal or persistent perinatal depressive symptoms sent fewer SMS messages during the study period than their counterparts without depression.

**Conclusions:**

Prevalence of elevated perinatal depressive symptoms was high in this cohort of Kenyan women. Our findings highlight the importance of screening perinatal women for experiences of symptoms of depression as well as abuse. Differences in messaging frequency between women with vs. without depressive symptoms presents an opportunity to provide more tailored support for those perinatal depression.

**Supplementary Information:**

The online version contains supplementary material available at 10.1186/s12884-022-05039-6.

## Background

Perinatal mood disorders, particularly depression, are associated with adverse maternal and infant outcomes and are an urgent global public health concern [1, 2]. Perinatal depression is broadly defined as an episode of major depression or depressive symptoms that occur during pregnancy and/or within the first year following childbirth [1]. A recent systematic review found that worldwide at least 17.2% of pregnant women and 13.1% of postpartum women suffer from perinatal depression [3]. Prevalence is even higher in low- and middle-income countries (LMICs) where approximately 25.8% of women have antenatal depression and 19.7% experience postpartum depression [4]. Risk factors for perinatal depression in LMICs include experiencing intimate partner violence, lower maternal educational opportunity, lower socioeconomic status, undesired pregnancy, single/unmarried status at time of pregnancy, and less social support [1, 2, 4, 5]. In studies conducted in Kenya, rates of maternal depression were higher in adolescents and women living with HIV (WLWH) [2]. Antenatal depression is the strongest risk factor for postpartum depression, with 54% of women with postpartum depression reporting depressive symptoms prior to childbirth [2, 4].

Research has consistently demonstrated an association between perinatal depression and adverse health outcomes for mothers and infants [4]. In particular, perinatal depression has been associated with preterm birth, intrauterine growth restriction, prolonged labor, low birth weight, persistent perinatal depression, and even maternal mortality by suicide [4, 6]. Recent studies suggest that subclinical depressive symptoms, i.e., symptoms consistent with depression that do not meet diagnostic criteria for major depressive disorder (MDD), have a similar impact as clinical depression/MDD on maternal and infant health outcomes [7]. Compared to infants of non-depressed mothers, infants of depressed mothers appear to have poorer physical and neurocognitive development and a higher likelihood of diarrheal, febrile, and other infectious illnesses [4]. One study amongst mothers in Nairobi, Kenya demonstrated how postpartum depression conferred a higher risk of non-exclusive breastfeeding and underweight infants [8]. While previous studies have characterized the consequences of perinatal depression for mothers and infants, there remains a paucity of literature from Sub-Saharan Africa, and particularly from Kenya, examining opportunities and interventions to mitigate maternal and infant morbidity and mortality associated with perinatal depression.

Identifying pregnant women with antenatal depression and those at high risk for developing postpartum depression provides a potential opportunity to intervene early, engage in therapeutic care, and reduce adverse health outcomes for women and their infants. Studies have shown that psychoeducational interventions initiated in the third trimester or early postpartum period can reduce prevalence and severity of postpartum depression, reduce neonatal mortality and improve overall infant growth and development [9]. In LMICs, perinatal depression often goes underrecognized and untreated, in part due to lack of mental health resources [4]. Even if depressive symptoms are recognized and mental health services are available, multiple challenges associated with pregnancy, motherhood, and stigma prohibit women from being able to seek treatment. Novel approaches are needed to enhance the capacity of the healthcare system to identify and care for women with perinatal depression in general, but particularly in places with a scarcity of mental health resources. One such potential solution to improve recognition, support for and treatment of perinatal depression in LMICs is employment of mobile health (mHealth) technologies [10]. mHealth encompasses the use of mobile and wireless technologies, including but not limited to voice and short messaging service (SMS), global positioning system (GPS), telecommunication networks, and Bluetooth technology, to support medical and public health practices [11].

While mHealth holds potential for improving perinatal health outcomes, most studies implementing mHealth interventions have been conducted in high-income countries [12–15]. Yet, mHealth interventions are a viable solution to connect healthcare workers with patients in LMICs, particularly in Kenya where at least 80% of Kenyans have their own mobile phone and there is an average of 2.4 mobile phones per household according to a 2011 national survey [16]. A first step in assessing mHealth utility in Kenya may be understanding how participants with depressive symptoms interact with a perinatal SMS messaging platform, a common mHealth intervention meant to be cost saving and elicit behavioral change. The aim of this study is to describe the prevalence of perinatal depression, assess correlates of depression, and evaluate the impact of maternal depression on infant outcomes in a cohort of Kenyan women participating in an SMS communication program. In addition, we sought to investigate the engagement of women living with depression with this perinatal mHealth intervention designed to improve maternal and infant health outcomes.

## Methods

### Study design

This study utilizes data collected from a prospective pilot cohort study, known as Mobile Solutions for Women, Adolescents, and Children’s Health: Neonate (Mobile WACh NEO Pilot). The Mobile WACh NEO Pilot study, designed to support maternal and infant outcomes by promoting facility delivery, infant survival and family planning uptake, enrolled 800 pregnant women seeking antenatal care services from two public facilities in Kenya: Mathare North Health Centre (Nairobi County, peri-urban) or Rachuonyo Sub-County Hospital (Homa Bay County, rural) from December 2017 to January 2019. These two public facilities offer a wide range of services for antenatal, birth center and postpartum care as well as have a high volume of daily antenatal appointments (> 10 new mothers per day) and serve a large community of low-income women and babies at high risk of neonatal morbidity and mortality. The Nairobi site serves women living in a large urban slum, and the Western Kenya site serves women living in a low-income rural area.

Mobile WACh NEO Pilot participants received pre-programmed SMS messages from enrollment during pregnancy until 14 weeks postpartum. Mobile WACh NEO is designed for maximal impact on neonatal morbidity. Thus, this intervention was implemented at the time when women and their infants are most at risk for experiencing morbidity and mortality, i.e., the last ANC visit (30–36 weeks) to the first postpartum visit (often delayed to approximately 14 weeks postpartum) in order to support and augment perinatal care in this critical period. Content and frequency of pre-programmed messages were dependent upon the woman’s pregnancy status and were delivered in the participant’s preferred language and time of day. Message content was not altered based on depression status. These messages targeted specific actionable health outcomes and encouraged engagement with the nurse. Participants could communicate with the nurse via SMS at any time free of charge. Study nurses managed the bidirectional SMS communication and used national guidelines and local practice standards for the care of pregnant/postpartum women and their infants when responding to participants’ questions. Prior to the start of the study, nurses were trained in these study procedures by two OBGYN physicians (Drs. Kinuthia and Unger) and the study nurse coordinator (Brenda Wandika). These physicians and nurse were available at all times for consultation. In addition, a twice monthly review of all messages was performed by Dr. Unger, and a message review implemented with the team of nurses. Two interviews, one at the time of enrollment and one post-intervention, were conducted with participants to collect demographic, health outcomes, and study-related information.

### mHealth intervention

Mobile WACh NEO is a two-way SMS communication intervention designed to engage women with a health care worker at their local clinic with the aim of improving maternal and neonatal outcomes. Messages are personalized, behavioral theory based and action oriented specific to the time point in pregnancy or postpartum. Participants are encouraged to reply to all messages they receive and initiate their own spontaneous messages throughout the study. A study nurse managed SMS communication with participants.

Schematic 1 SMS messaging content and frequency

**Table:** 

***Period***	***Pregnancy*** *(Enrollment – Delivery)*	***Early neonatal period*** *(Delivery- 4 weeks infant age)*	***Postnatal period*** *(4 weeks – 12 weeks infant age)*
***SMS frequency***	Weekly	Daily × 1 week ➔ Every other day × 3 weeks	Twice a week
***Topics***	Birth preparation	Infant and maternal health evaluations	Infant health and family planning

### Study population

Pregnant women seeking ANC services from the two sites were recruited to participate in the Mobile WACh NEO Pilot intervention. This source population encompassed both rural and peri-urban areas, an ethnically diverse population, and areas with generally low socioeconomic status and high neonatal mortality. Pregnant women were eligible if they had daily access to a mobile phone, were ≥ 14 years of age, and were between 30 and 36 weeks gestation. If a woman was not sufficiently literate but had access to a partner or family member whom she would be comfortable having read her messages, she was eligible for the study. Pregnant women were recruited by community health workers who introduced the study to potential participants, answered questions, and invited women to participate. We introduced the study to all women attending ANC visits at the two clinics between December 2017 and May 2018, and using a convenience sample, we recruited those who agreed to participation and met inclusion criteria. Women were recruited and enrolled on the same day at these two facilities. It was emphasized that participation was completely voluntary and would not in any way affect their antenatal, postnatal, or infant care services. Women who were referred and willing to participate were given a screening questionnaire in order to assess eligibility. Oral consent was obtained for participation in screening. Eligible women who agreed to participate and receive SMS messages provided written informed consent and were entered into the Mobile WACh system along with their preferences for SMS message delivery. Eligible women who chose not to participate were asked their reasons for non-participation, with responses recorded in the screening questionnaire. Women who screened positive for depressive symptoms were referred to available mental health resources, which was the same referral process utilized by the ANC clinics for women not included in the study and typically involved a social worker. Researchers were blinded to a woman’s depression status until after the study concluded; thus, referrals were made within the standard process of the clinical site without interference from the study itself.

### Data collection

Women were followed during pregnancy and for 14 weeks postpartum. Participants were administered a standardized questionnaire at enrollment and one follow-up visit (at 14 weeks postpartum) using a tablet-based system (Open Data Kit, ODK) [17]. Exit surveys were conducted either in-person or via telephone, but data on depressive symptoms was not gathered during the phone exit surveys. We collected patient information including questions pertaining to demographics, medical history, experience with SMS and technology, and depression. The Abuse Assessment Screen (AAS) was used to evaluate maternal experience of violence based on participant reports of experiencing physical abuse during the current pregnancy [18]. The AAS has been used as a measure of intimate partner violence in previous studies but is not specific to abuse inflicted by a sexual partner [19]. Undesired pregnancy was defined as the mother reporting she did not want to have a/another baby at the time of becoming pregnant with the current pregnancy. History of miscarriage was a binary variable consisting of women who had reported at least one spontaneous abortion prior to current pregnancy. SMS communication was collected continuously in the Mobile WACh platform throughout the entirety of the study period.

#### Infant and delivery information

Preterm births were defined as delivery prior to 37 weeks estimated gestational age. The type of delivery was classified as either a vaginal delivery, planned Cesarean section (C-section), or unplanned/emergency C-section, based on self-report. Infant morbidity was defined as a mother affirming her child had been to any clinic/hospital for any illness after delivery but before follow-up at 14 weeks or her child had been admitted to the hospital after delivery. Infant mortality was based on maternal report of infant death.

#### Depression status

The prevalence of depressive symptoms was assessed by dichotomizing self-reported participant Edinburgh Postnatal Depression Scale (EPDS) scores into: < 10, categorized as “no depression” and ≥ 10, categorized as “depression”. For the purposes of this study, we stratified women in this cohort into four patterns of depressive symptoms: 1) antenatal depression (EPDS score ≥ 10 at enrollment), 2) postpartum depression (EPDS score ≥ 10 at follow-up), 3) persistent perinatal depression (EPDS score ≥ 10 at both time-points) and 4) any perinatal depression (EPDS score ≥ 10 at any time-point) [3, 20]. Therefore, women with postpartum depression could represent new-onset postpartum depression (incident depression) or continuation of depression from the antepartum period [20], while any perinatal depression could represent women with antenatal depression only, postpartum depression only, or persistent perinatal depression. Any perinatal depression is meant to account for the fact that depressive symptoms during the perinatal period are dynamic and have historically not been well-characterized longitudinally but rather only in the antenatal or postpartum periods [3].

#### SMS message data

All SMS messages sent to and received from participants were recorded in the Mobile WACh messaging platform. The sender of the message was classified as the system, the participant, or the nurse. For the purposes of this analysis, only messages that originated from the participants were used to assess level of interaction with the two-way messaging system. Character counts of messages were documented based on the original text messages sent. Participant SMS messaging by participants was categorized into ever having sent ≥1 SMS during the study period vs. never having sent an SMS. Messages whose character count was ≥10 characters were classified as long SMS messages, in an effort to capture conversational messages rather than messages that simply acknowledged receipt of system message. If a participant sought advice from the study nurse, this initiative was considered a nurse consult in this study.

### Statistical analyses

Statistical analysis was performed using the program R studio version 1.2.5001 (Boston, 2019). All tests were considered statistically significant at an alpha level of 0.05.

Descriptive analyses for baseline sociodemographic and obstetric characteristics of women who completed a second EPDS at follow-up were conducted.

Correlates of depression were identified using univariable and multivariable generalized estimating equation (GEE) with Poisson link clustered by facility, with exchangeable correlation structure and robust standard errors. Poisson regression was used to generate effect estimates that could be interpreted as relative risk despite common outcomes. GEE was used to account for similarities by site. Multivariable models were run to control for potential confounders, determined a priori based on literature review: adolescent age (< 20), marital status, education level, employment status, HIV status, monthly income, number of living children, primigravid status, history of miscarriage, experience of abuse during pregnancy, pregnancy desire, and previous family planning use. The main analysis identified correlates of any perinatal depression and secondary analyses were conducted on correlates of each pattern of depression. For the continuous variable of monthly income, results were interpreted as the relative risk of having the depression comparing mothers with 1000 KSh (~ 10 USD) difference in monthly household income. The relative risk for the variable “living children” was interpreted as relative risk of depression associated with one additional living child.

Association of antenatal depression as a predictor of infant outcomes of preterm birth, infant morbidity, and infant mortality was analyzed using GEE with Poisson link, clustered by facility, with exchangeable correlation structure and robust standard errors. Univariable and multivariable regressions were run. Confounders in multivariable analyses were chosen a priori from literature review and included adolescence, marital status, education level, employment status, abuse during pregnancy, HIV status, monthly income, distance from clinic, type of delivery, location of delivery, complications during pregnancy, and complications during delivery.

We evaluated association of depression with several measures of participant engagement with the SMS platform: a binary outcome of any SMS message sent over the study period; the total number of SMS messages sent; and the total number of long SMS messages (≥10 characters). Each measure of engagement was compared between women with and without depression using univariable and multivariable GEE with Poisson link, clustering by facility, exchangeable correlation structure and robust standard errors. Only antenatal and persistent perinatal depression were included as depression patterns so that depressive symptoms preceded the outcomes of interest. All women with persistent perinatal depression were included in the antenatal depression cohort given the definitions described above.

Chi-squared tests were used to assess differences in study nurse consultation between women with and without depression. Again, only antenatal and persistent perinatal depression were included as these depression patterns preceded the outcomes of interest. Fisher’s exact tests were used when sample sizes were too small to get accurate results from Chi-squared tests.

## Results

### Participant characteristics

**Fig. 1:**
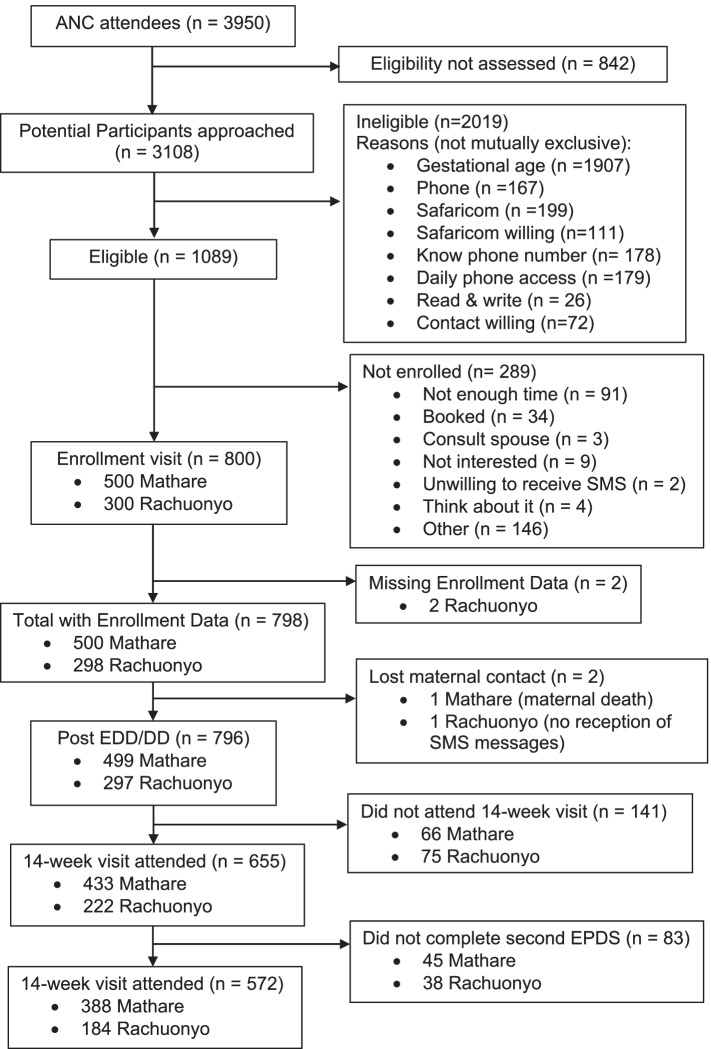
Diagram to show recruitment and enrollment of study participants

Of a total of 800 participants, two participants had missing enrollment information due to data entry error and were excluded (Fig. 1). There were 143 (18%) women lost-to-follow up and 83 women without a follow-up EPDS score because their exit interview was performed by phone, and EPDS was excluded in phone follow-up. Thus, a total of 226 participants (28.3%) did not have outcomes for postpartum depression and were excluded from the analysis (Fig. 1).

The attrition rate in Rachuonyo is noted to be higher than at Mathare Health Center due to the more rural environment served by Rachuonyo as well as intermittent health care worker strikes at this facility (Fig. 1). The same strikes did not occur at Mathare, which is run by the city council. Women residing around Rachuonyo were more likely to travel to other places to have their babies than women residing around Mathare.

Table 1 displays baseline characteristics of the 572 participants with two EPDS scores. The median age of women in the cohort was 25 years and 10.2% of participants were adolescents age < 20. Approximately 11.3% of participants were not married nor cohabitating and 8.2% had less than a primary level of education. Most women were multigravidas. Approximately 34.2% of participants reported the pregnancy was undesired, i.e., the current pregnancy occurred despite a participant’s desire not to have a/another baby. On average, participants had one living child.

**Table 1:** Participant Baseline Characteristics

Variables	Total Cohort (***n*** = 572) *n* (%) or median (IQR)
**Age in years**	25 (22–29)
**Adolescents**	58 (10.2%)
**Marital Status**	
Not Married/Cohabitating	64 (11.3%)
**Education level**	
Less than primary education	47 (8.2%)
**Employment Status**	
Unemployed	394 (68.9%)
**Primigravida**	194 (33.9%)
**Number of Living Children**	1 (0–2)
**History of Miscarriage**	65 (11.4%)
**Undesired Pregnancy**	195 (34.2%)
**Never Used Family Planning**	160 (28.0%)
**Median monthly household income in KSh**	10,000 (6000–14,000)
**Experiencing abuse during pregnancy**	19 (3.3%)
**Living with HIV**	36 (6.3%)
**Distance from home to clinic ≥ 30 min**	358 (62.7%)
**Share the phone with someone**	81 (14.2%)

### Prevalence of depression

Among the 572 participants with two completed EPDS surveys, 188 (32.9%) of participants reported depressive symptoms at some time during the perinatal period, 163 (28.5%) reported antenatal symptoms, 52 (9.1%) reported postnatal symptoms, and 27 (4.7%) reported persistent symptoms (Table 2).

**Table 2:** Correlates of Perinatal Depression

*Correlates*	***Any Perinatal Depression*** *n (%) or Median (IQR)*		*Relative Risk (95% CI)*	*p-value*	*Adjusted Relative Risk*^a^ *(95% CI)*	*p-value*
	*Yes* *188 (32.9%)*	*No* *384 (67.1%)*				
Adolescent	22 (12.0%)	36 (9.4%)	1.10 (0.86, 1.39)	0.45	1.01 (0.79, 1.28)	0.96
Not Married/Cohabitating	26 (14.3%)	38 (9.9%)	1.18 (0.88, 1.58)	0.26	1.00 (0.96, 1.05)	0.86
Less than primary education	15 (8.0%)	32 (8.3%)	1.02 (0.99, 1.05)	0.13	1.04 (0.94, 1.15)	0.43
Unemployed	127 (67.6%)	267 (69.5%)	1.07 (0.93, 1.23)	0.34	1.03 (0.95, 1.11)	0.50
Primigravida	63 (33.5%)	131 (34.1%)	1.09 (0.95, 1.24)	0.21	1.14 (1.04, 1.24)	0.005*
Living Children^b^	1 (0–2)	1 (0–2)	1.01 (0.95, 1.06)	0.83	1.05 (0.99, 1.10)	0.08
History of Miscarriage	16 (8.5%)	49 (12.8%)	0.81 (0.61, 1.08)	0.15	0.81 (0.74, 0.88)	< 0.0001*
Undesired pregnancy	83 (44.1%)	112 (29.3%)	1.30 (1.11, 1.52)	0.001*	1.23 (1.13, 1.35)	< 0.0001*
Never Used Family Planning	58 (30.9%)	102 (26.6%)	1.00 (0.98, 1.02)	0.99	0.98 (0.79, 1.22)	0.87
Monthly Income (in KSh) ^c^	8000 (2000–12,000)	10,000 (8000–14,000)	1.00 (0.99, 1.01)	0.98	1.00 (0.99, 1.01)	0.87
Abuse during pregnancy	10 (5.3%)	9 (2.3%)	1.73 (1.57, 1.90)	< 0.0001*	1.76 (1.64, 1.90)	< 0.0001*
Living with HIV	13 (7.0%)	23 (6.0%)	0.88 (0.43, 1.81)	0.73	0.86 (0.48, 1.54)	0.62
*Correlates*	***Antenatal Depression*** *n (%) or Median (IQR)*		*Relative Risk (95% CI)*	*p-value*	*Adjusted Relative Risk*^a^ *(95% CI)*	*p-value*
	*Yes* *163 (28.5%)*	*No* *409 (71.5%)*				
Adolescent Age	21 (13.2%)	37 (9.1%)	1.19 (0.76, 1.88)	0.44	1.04 (0.71, 1.52)	0.85
Not Married/Cohabitating	24 (15.3%)	40 (9.8%)	1.24 (0.90, 1.71)	0.18	1.10 (1.00, 1.21)	0.04*
Less than primary education	13 (8.0%)	34 (8.3%)	1.04 (0.97, 1.10)	0.31	0.99 (0.94, 1.05)	0.74
Unemployed	113 (69.3%)	281 (68.7%)	1.19 (1.18, 1.20)	< 0.001*	1.09 (1.08, 1.11)	< 0.0001*
Primigravida	55 (33.7%)	139 (34.0%)	1.12 (0.94, 1.35)	0.21	1.21 (1.02, 1.42)	0.03*
Living Children^b^	1 (0–2)	1 (0–2)	1.01 (0.95, 1.07)	0.79	1.08 (1.08, 1.08)	< 0.0001*
History of Miscarriage	16 (9.8%)	49 (12.0%)	0.95 (0.84, 1.08)	0.41	0.94 (0.90, 0.99)	0.03*
Undesired pregnancy	73 (44.8%)	122 (30.0%)	1.28 (1.16, 1.41)	< 0.0001*	1.17 (1.14, 1.20)	< 0.0001*
Never Used Family Planning	53 (32.5%)	107 (26.2%)	1.04 (0.96, 1.13)	0.36	1.00 (0.90, 1.12)	0.97
Monthly Income (in KSh) ^c^	6000 (1500–10,500)	10,000 (8000–15,000)	0.99 (0.99, 0.99) ^d^	< 0.0001*	0.99 (0.99, 0.99) ^e^	< 0.0001*
Abuse during pregnancy	9 (5.5%)	10 (2.4%)	1.81 (1.59, 2.05)	< 0.0001*	1.79 (1.65, 1.94)	< 0.0001*
Living with HIV	12 (7.4%)	24 (5.9%)	0.90 (0.50, 1.61)	0.71	0.83 (0.53, 1.31)	0.43
*Correlates*	***Postpartum Depression*** *n (%) or Median (IQR)*		*Relative Risk (95% CI)*	*p-value*	*Adjusted Relative Risk*^a^ *(95% CI)*	*p-value*
	*Yes* *52 (9.1%)*	*No* *52 (90.9%)*				
Adolescent Age	4 (8.0%)	54 (10.4%)	0.74 (0.23, 2.39)	0.61	0.79 (0.32, 1.99)	0.62
Not Married/Cohabitating	6 (11.8%)	58 (11.3%)	0.99 (0.80, 1.22)	0.89	0.81 (0.51, 1.29)	0.38
Less than primary education	6 (11.5%)	41 (7.9%)	1.47 (1.35, 1.60)	< 0.0001*	1.51 (1.29, 1.78)	< 0.0001*
Unemployed	34 (65.4%)	360 (69.2%)	0.93 (0.49, 1.76)	0.83	0.97 (0.62, 1.52)	0.89
Primigravida	14 (26.9%)	180 (34.6%)	0.78 (0.76, 0.80)	< 0.0001*	1.33 (1.29, 1.37)	< 0.0001*
Living Children^b^	1 (0–2)	1 (0–2)	1.22 (1.15, 1.29)	< 0.0001*	1.18 (1.00, 1.40)	0.05
History of Miscarriage	7 (13.5%)	58 (11.2%)	1.25 (0.40, 3.88)	0.70	0.84 (0.32, 2.25)	0.74
Undesired pregnancy	21 (40.4%)	174 (33.6%)	1.18 (0.84, 1.67)	0.34	1.07 (0.68, 1.70)	0.77
Never Used Family Planning	13 (25.0%)	147 (28.3%)	0.79 (0.48, 1.30)	0.35	0.88 (0.46, 1.69)	0.70
Monthly Income (in KSh) ^c^	8000 (2000–16,000)	10,000 (6000–14,000)	1.01 (0.99, 1.02)	0.30	1.01 (1.00, 1.02)	0.05*
Abuse during pregnancy	2 (3.8%)	17 (3.3%)	1.24 (0.94, 1.64)	0.12	1.53 (1.13, 2.07)	0.006*
Living with HIV	5 (9.8%)	31 (6.0%)	1.39 (0.59, 3.27)	0.45	1.45 (0.85, 2.47)	0.17
*Correlates*	***Persistent Perinatal Depression*** *n (%) or Median (IQR)*		*Relative Risk (95% CI)*	*p-value*	*Adjusted Relative Risk*^a^ *(95% CI)*	*p-value*
	*Yes* *27 (4.7%)*	*No* *545 (95.3%)*				
Adolescent Age	3 (12.0%)	55 (10.2%)	1.02 (0.75, 1.40)	0.88	0.95 (0.86, 1.05)	0.30
Not Married/Cohabitating	4 (15.4%)	60 (11.1%)	1.16 (0.85, 1.59)	0.35	1.34 (1.05, 1.71)	0.02*
Less than primary education	4 (14.8%)	43 (7.9%)	1.89 (1.74, 2.06)	< 0.0001*	1.39 (0.91, 2.10)	0.12
Unemployed	20 (74.1%)	374 (68.6%)	1.58 (1.32, 1.89)	< 0.0001*	1.36 (1.06, 1.75)	0.02*
Primigravida	6 (22.2%)	188 (34.5%)	0.76 (0.74, 0.78)	< 0.0001*	1.76 (1.29, 2.40)	0.0004*
Living Children^b^	2 (0.5–3)	1 (0–2)	1.32 (1.24, 1.41)	< 0.0001*	1.39 (1.25, 1.54)	< 0.0001*
History of Miscarriage	7 (25.9%)	58 (10.6%)	2.59 (2.04, 3.28)	< 0.0001*	1.46 (1.00, 2.13)	0.05*
Undesired pregnancy	11 (40.7%)	184 (33.9%)	1.00 (0.95, 1.04)	0.87	0.75 (0.66, 0.86)	< 0.0001*
Never Used Family Planning	8 (29.6%)	152 (27.9%)	0.84 (0.63, 1.13)	0.25	0.95 (0.76, 1.19)	0.67
Monthly Income (in KSh)^c^	2000 (1500–6600)	10,000 (6500–14,000)	0.96 (0.95, 0.97)	< 0.0001*	0.97 (0.96, 0.98)	< 0.0001*
Abuse during pregnancy	1 (3.7%)	18 (3.3%)	1.35 (0.86, 2.14)	0.20	1.51 (0.99, 2.30)	0.05
Living with HIV	4 (15.4%)	32 (5.9%)	1.75 (1.50, 2.03)	< 0.0001*	1.43 (1.34, 1.52)	< 0.0001*

^a^Adjusted for all other correlates listed in table

^b^This relative risk is given as the risk of having any perinatal depression comparing a mother with one additional living child to a mother with a certain number of living children

^c^This relative risk is given as the risk of having any perinatal depression comparing a mother with an additional 1000 KSh in monthly household income to a mother of a certain monthly household income

^d^Taken to the ten-thousandth place, this relative risk and corresponding CI is 0.9889 (0.9885, 0.9893)

^e^Taken to the ten-thousandth place, this adjusted relative risk and corresponding CI is 0.9917 (0.9912, 0.9923)

Table 2 demonstrates correlates of perinatal depression.

#### Any perinatal depression correlates

The risk of any perinatal depression was higher in women with undesired pregnancies (RR = 1.30; 95% CI: 1.11, 1.52), and in those who experienced abuse during pregnancy (RR = 1.73; 95% CI: 1.57, 1.90). All associations remained significant in multivariable analyses (Table 2).

#### Antenatal depression correlates

An elevated risk of antenatal depression was observed in women with undesired pregnancies (RR =1.28; 95% CI 1.16, 1.41), women who experienced abuse during pregnancy (RR = 1.81; 95% CI: 1.59, 2.05), women with lower monthly incomes (RR = 0.99; 95% CI: 0.99, 0.99), and women who were unemployed (RR 1.19, 95% CI: 1.18,1.20). All associations remained significant in multivariable analyses (Table 2).

#### Postpartum depression correlates

A higher risk of postpartum depression was associated with women with less than a primary level of education (RR = 1.47; 95% CI: 1.35, 1.60) and women with more living children (RR = 1.22; 95% CI: 1.15, 1.29). In multivariate analysis, the association remained significant for women with less than primary education (Table 2). A lower risk of postpartum depression was observed in women who were primigravida (RR = 0.78; 95% CI: 0.76, 0.80), though this association reversed in the multivariable analysis (aRR = 1.33; 95% CI: 1.29, 1.37).

#### Persistent perinatal depression correlates

There was a higher risk of persistent perinatal depression associated with women with less than a primary education (RR = 1.89; 95% CI: 1.74, 2.06), women who were unemployed (RR = 1.58; 95% CI: 1.32, 1.89), women with an additional living child (RR = 1.32; 95% CI 1.24, 1.41), women with a history of miscarriage (RR = 2.59; 95% CI: 2.04, 3.28), women with a lower monthly income (RR = 0.96; 95% CI: 0.95, 0.96), and women living with HIV (RR = 1.75; 95% CI: 1.50, 2.03). All these associations remained significant in multivariable analyses except the risk associated with less than a primary education (Table 2). A lower risk of persistent perinatal depression was observed in women who were primigravida (RR = 0.76; 95% CI: 0.74, 0.78), though this association reversed in the multivariable analysis (aRR = 1.76; 95% CI: 1.29, 2.40).

### Antenatal depression and infant outcomes

A total of 271 infants were hospitalized or were attended at the clinic for illness after birth for a morbidity rate of 474 per 1000 births. One hundred forty-two infants were preterm (25.0%) and 18 (3.2%) infant deaths occurred (Table 3) for an infant mortality rate of 31 per 1000 live births. Antenatal depression was associated with an increased risk of infant morbidity in both the univariate (RR = 1.12; 95% CI: 1.11, 1.13) and multivariate analyses (aRR = 1.03; 95% CI: 1.03, 1.03). There was no significant association between antenatal depression and preterm birth in either the univariate or multivariate analyses. Antenatal depression was associated with a decreased relative risk of infant mortality (RR = 0.77; 95% CI: 0.65, 0.91). Multivariate analysis of the association between antenatal depression and infant mortality could not be determined due to small sample size.

**Table 3:** Association between antenatal depression and infant outcomes

*Outcome (n)*	*Proportion with Outcome (n; %)*	*Relative Risk (95% CI)*	*p-value*	*Adjusted Relative Risk (95% CI)*^a^	*p-value*
**Infant Morbidity**		**271 (47.4%)**			
Antenatal depression (*n* = 163)	84 (51.5%)	1.12 (1.11, 1.13)	< 0.0001*	1.03 (1.03, 1.03)^b^	< 0.0001*
No antenatal depression (*n* = 409)	187 (45.7%)				
**Preterm Birth**		**142 (25.0%)**			
Antenatal depression (*n* = 162)	41 (25.3%)	1.22 (0.75, 1.99)	0.42	1.32 (0.89, 1.97)	0.17
No antenatal depression (*n* = 406)	101 (24.9%)				
**Infant Mortality**		**18 (3.1%)**			
Antenatal depression (*n* = 163)	5 (3.1%)	0.77 (0.65, 0.91)	0.002*	–	–
No antenatal depression (*n* = 409)	13 (3.2%)				

^a^Adjusted for adolescence, marital status, education level, employment status, abuse during pregnancy, HIV status, monthly income, primigravida status, distance from clinic, type of delivery, location of delivery, complications during pregnancy, and complications during delivery

^b^Taken to thousandth place, the adjusted relative risk and corresponding CI is 1.030 (1.029, 1.031)

### Association between depression and SMS messaging level

We evaluated the association between perinatal depression and SMS messaging at different times in the perinatal period.

All 572 participants received preprogrammed messages from the SMS system. Over 90% of women sent an SMS message at some time (Table 4). The likelihood of ever sending an SMS message to the messaging system was significantly lower for mothers with antenatal depression as compared to mothers who did not have antenatal depression (aRR = 0.97; 95% CI: 0.97, 0.98). There was no significant association between persistent perinatal depression and likelihood of ever sending an SMS.

**Table 4:** Association between perinatal depression and SMS messaging patterns

**ANTENATAL DEPRESSION**					
**Ever vs. never sending an SMS message**					
*Presence of antenatal depression (n)*	*Proportion who ever sent an SMS (n (%)*	*Relative Risk (95% CI)*	*p-value*	*Adjusted Relative Risk*^a^ *(95% CI)*	*p-value*
Yes (*n* = 163)	150 (92.0%)	0.99 (0.98, 1.00)	0.05*	0.97 (0.97, 0.98)	< 0.0001*
No (*n* = 409)	384 (93.9%)				
**Total participant SMS sent**					
*Presence of antenatal depression (n)*	*Total SMS Count* *Median (IQR)*	*Relative Risk (95% CI)*	*p-value*	*Adjusted Relative Risk*^a^ *(95% CI)*	*p-value*
Yes (*n* = 163)	14 (5–38)	0.77 (0.72, 0.82)	< 0.0001*	0.91 (0.87, 0.96)	< 0.0001*
No (*n* = 409)	25 (8–44)				
**Total participant long SMS sent**					
*Presence of antenatal depression (n)*	*Long SMS Count* *Median (IQR)*	*Relative Risk (95% CI)*	*p-value*	*Adjusted Relative Risk*^a^ *(95% CI)*	*p-value*
Yes (*n* = 163)	12 (3–28)	0.81 (0.79, 0.84)	< 0.0001*	0.92 (0.89, 0.96)	< 0.0001*
No (*n* = 409)	19 (6–34)				
**PERSISTENT PERINATAL DEPRESSION**					
**Likelihood of ever versus never sending an SMS message**					
*Presence of persistent depression (n)*	*Proportion who ever sent an SMS (n (%)*	*Relative Risk (95% CI)*	*p-value*	*Adjusted Relative Risk*^a^ *(95% CI)*	*p-value*
Yes (*n* = 27)	25 (92.6%)	1.00 (0.93, 1.07)	0.91	0.96 (0.91, 1.00)	0.08
No (*n* = 545)	509 (93.4%)				
**Ratio of mean participant SMS counts**					
*Presence of persistent depression (n)*	*Total SMS Count* *Median (IQR)*	*Relative Risk (95% CI)*	*p-value*	*Adjusted Relative Risk*^a^ *(95% CI)*	*p-value*
Yes (*n* = 27)	15 (8–35)	0.74 (0.54, 1.01)	0.05	0.81 (0.70, 0.94)	0.005*
No (*n* = 545)	22 (7–44)				
**Ratio of mean participant long SMS messages sent**					
*Presence of persistent depression (n)*	*Long SMS Count* *Median (IQR)*	*Relative Risk (95% CI)*	*p-value*	*Adjusted Relative* Risk^a^ *(95% CI)*	*p-value*
Yes (*n* = 27)	13 (7–31)	0.78 (0.62, 0.97)	0.03*	0.86 (0.76, 0.98)	0.02*
No (*n* = 545)	17 (5–33)				

^a^Adjusted for self-reported SMS behaviors including participant response rate to received messages, physical sharing of phone, phone access, SMS as participants’ primary mode of communication, typical SMS use, and difficulty receiving SMS messages from the system

The median number of total SMS messages sent by participants over the entire study period and the median number of long SMS messages sent by participants during the study were analyzed to assess association of depressive symptoms with level of engagement with the program. Mothers with antenatal depression sent significantly fewer total SMS messages (aRR = 0.91; 95% CI: 0.87, 0.96) and fewer long SMS messages (aRR = 0.92; 95% CI: 0.89, 0.96) as compared to their counterparts without depression (Tables 4). Mothers with persistent perinatal depression sent fewer total SMS messages (aRR = 0.81; 95% CI: 0.70, 0.94) and fewer long SMS messages (aRR = 0.86; 95% CI: 0.76, 0.98) (Table 4).

### Association between depression and nurse consultation

#### Nurse consultations regarding general questions

Among the 572 women with complete depression data, 423 (74.0%) women reported that they ever consulted the study nurse. There were no significant differences between women with antenatal depression and those with persistent perinatal depression as compared to their non-depressed counterparts who consulted the project nurse at any time (Table 5). All participants (100%) who initiated a consult confirmed the consult was helpful regardless of depression pattern.

**Table 5:** Nurse consultation among participants with vs. without depression

*Reason for Consult*	*Depression Type (n)*	*Proportion of Consults for this Reason (n; %)*	*p-value*
Consult for Any Reason	Never Any Perinatal Depression (*n* = 384)	287 (74.7%)	Reference
	Antenatal Depression (*n* = 163)	118 (72.4%)	0.60
	Persistent Perinatal Depression (*n* = 27)	16 (59.3%)	0.10
All Participants who Ever Consulted Nurse			
Pregnancy Questions/Concern	Never Any Perinatal Depression (*n* = 287)	249 (86.8%)	Reference
	Antenatal Depression (*n* = 118)	109 (92.4%)	0.20
	Persistent Perinatal Depression (*n* = 16)	12 (75.0%)	0.30
Postpartum Questions/Challenges	Never Any Perinatal Depression (*n* = 287)	140 (48.8%)	Reference
	Antenatal Depression (*n* = 118)	63 (53.4%)	0.50
	Persistent Perinatal Depression (*n* = 16)	8 (50.0%)	1.00
Family Planning	Never Any Perinatal Depression (*n* = 287)	167 (58.2%)	Reference
	Antenatal Depression (*n* = 118)	57 (48.3%)	0.09
	Persistent Perinatal Depression (*n* = 16)	7 (43.8%)	0.40
Infant Health Questions/Concerns	Never Any Perinatal Depression (*n* = 287)	249 (86.8%)	Reference
	Antenatal Depression (*n* = 118)	90 (76.3%)	0.01*
	Persistent Perinatal Depression (*n* = 16)	13 (81.2%)	0.50
Subgroup of Participants with Ill Infants who Ever Consulted Nurse			
Infant Health Consult	Never Any Perinatal Depression (*n* = 167)	54 (32.3%)	Reference
	Antenatal Depression (*n* = 79)	15 (19.0%)	0.04*
	Persistent Perinatal Depression (*n* = 8)	2 (25.0%)	1.00

Fewer mothers with antenatal depression consulted the study nurse regarding infant health concerns as compared to women without depression (76.3% vs. 86.8%, *p* = 0.01) (Table 5). Otherwise, mothers with antenatal depression consulted the study nurse regarding the remaining consult categories (pregnancy questions, postpartum questions, and family planning questions) at a similar rate as mothers who never had depression (Table 5). There was no significant difference in any reason for consulting the study nurse when comparing women with persistent perinatal depression to women who never had perinatal depression (Table 5).

#### Nurse consultations regarding infant’s illness

Two hundred seventy-one women reported on the follow-up survey that their infants had had an illness that necessitated a clinic/hospital visit or hospital admission after birth. Of the 271 mothers with ill infants, 253 mothers reported whether they contacted the study nurse about their infant’s illness. Mothers with antenatal depression were less likely to consult the study nurse about their ill infant than mothers without depression (19.0% vs. 32.3%, *p* = 0.04) (Table 5). There was no significant difference in the proportion of mothers with persistent perinatal depression and mothers who never had depression who consulted the nurse about their ill infant (Table 5). All participants (100%) who made consults regarding their infant’s illness reported the consult was helpful.

## Discussion

This prospective study of pregnant women in Kenya demonstrated a relatively high prevalence of any perinatal depression and antenatal depression. This is one of few studies to evaluate depressive symptoms longitudinally in both the antepartum and postpartum periods. In this cohort, more women had any perinatal depression (32.9%), which is mainly driven by the large proportion of women who had antenatal depression (28.5%) while only 9.1% of women had postpartum depression, which is lower than anticipated given previous studies. One study of pregnant Sudanese women established a similar postpartum depression prevalence of 9.2% at 12 weeks postpartum [21], but most African studies have reported a higher prevalence of postpartum depression [22]. For instance, the estimated postpartum depression rate in Kenya has been cited as ranging from 13 to 18.7% in previous studies [2, 8, 22]. Neighboring countries of Tanzania and Ethiopia demonstrated similar postpartum depression rates, 12 and 20.9%, respectively, while Uganda was most notable for its 43% postpartum depression rate when using EPDS as the assessment tool [22]. Of note, 51.9% of women with postpartum depression in our study had antenatal depression. This is consistent with Gelaye et al.’s finding that a little more than half of women who have postpartum depression subsequently were found to have depressive symptoms present in the antenatal period [4]. Other studies have similarly demonstrated that antenatal depression is a significant risk factor for the development of depression in the postpartum period [2, 20]. Taken together, all these results highlight the importance of screening for depression in the entire perinatal period and emphasize the need to include the antepartum period in perinatal depression research, particularly given its impact on both maternal, pregnancy and infant outcomes.

It is evident that rates of perinatal depression are affected by psychosocial stressors and the broader social context of a woman’s life. Experiencing abuse was the strongest risk factor for perinatal depression. Other studies corroborate our findings and have demonstrated strong associations between abuse, particularly in the form of intimate partner violence, and increased risk of incident and persistent perinatal depression [2, 4, 20]. Consistent with other studies [4, 23, 24], women with less than a primary education had a significantly increased risk of postpartum and persistent perinatal depression. Participants living with HIV had an increased risk for persistent perinatal depression. The association between HIV and perinatal depressive symptoms has been well-documented in both the antenatal and postnatal periods [25, 26]. Finally, maternal unemployment was a significant risk factor for antenatal depression, with an even stronger association between maternal unemployment and persistent perinatal depression. Previous literature in high-income countries has provided evidence that maternal unemployment is a risk factor for perinatal depression, while a recent study in South Africa found a borderline association of antenatal depression associated with maternal unemployment [22, 27]. More studies are needed to elucidate this relationship.

Accessing prenatal care is often challenging for adolescent women for a variety of systemic reasons, including social stigma and fear of judgment and stigma by health care providers [28]. Surprisingly, adolescent age was not significantly associated with any increased risk of developing perinatal depression. One reason for this finding may be that we defined adolescents as women aged 14–19 in this study, consistent with the WHO definition of adolscence [29]. Thirty-eight (65.5%) of the 58 adolescents enrolled in this study were 19 years old. Previous studies examining the relationship between adolescence and perinatal depression often demonstrate an inverse relationship between age and depressive symptoms where adolescents ≤16 years old are more likely to have depressive symptoms than older adolescents ages 18–19 [30, 31]. If our sample had included a larger portion of younger adolescents, our results may have been more consistent with established literature.

Becoming pregnant without the intent of having a/another baby at some time was associated with significantly higher risk of any perinatal depression and antenatal depression, but not postpartum depression. Surprisingly, we found that having an undesired pregnancy was in fact associated with a decreased risk of persistent perinatal depression after adjusting for a priori confounders. There have been a plethora of studies suggesting that undesired pregnancies are associated with an increased risk of perinatal depression [23, 32–34], which is consistent with most of our findings. There is some evidence that undesired pregnancy is associated with depressive symptoms in the first trimester, with waning significance over time [23]. One U.S. study demonstrated that regardless of pregnancy intention, severity of depressive symptoms diminished in the postpartum period [33]. Additionally, our metric did not measure unintended pregnancies, which include both undesired and mistimed pregnancies, i.e., pregnancies occurring earlier than desired [20, 33]. This decision to separate undesired pregnancies from mistimed pregnancies may have confounded our results since desired and mistimed pregnancies were lumped together as one measure while undesired pregnancy was a separate measure. A systematic review of unintended pregnancies and perinatal depression found that unwanted pregnancies had a slightly higher association with perinatal depression than mistimed pregnancies [34]. Thus, our results may reflect either a true diminishment of depressive symptoms after childbirth despite initial lack of maternal desire for a/another child; a distortion by our inability to detect subclinical depressive symptoms in the postpartum period; and/or evidence of the complex relationship between depressive symptoms and maternal attitudes toward pregnancy.

In this cohort, a history of miscarriage was significantly associated with an increased risk of persistent perinatal depression but a decreased risk of any perinatal depression and antenatal depression on multivariate analyses. Having to deal with a previous pregnancy loss may fortify a woman’s capacity to handle the stress of, and even provide hope/enthusiasm for, a current pregnancy in the antenatal period. In the long term, the memory of a pregnancy loss in the face of a new maintained pregnancy with a subsequent live birth may predispose women to having a higher risk persistent perinatal depression if antenatal depression was present. These findings emphasize how crucial it is to longitudinally track maternal mood symptoms as maternal feelings and attitudes about pregnancy and motherhood change over time.

There is ample research that has documented the association between antenatal depression and increased likelihood of preterm birth [4, 5, 35]. Interestingly, we did not find any significant associations between antenatal depression and preterm birth. Additionally, antenatal depression was associated with a significant reduction in the risk of a woman’s infant dying in the immediate postpartum period in our univariate analysis. However, infant mortality was defined only up to 14 weeks of age, as compared to most research which extends mortality in children up to one year of age [36]. Due to this small sample size, an adequate multivariate analysis for an association between antenatal depression and infant mortality could not be conducted; therefore, this remains inconclusive in our study. Previous studies in rural Ghana and Ethiopia did not find any association with antenatal depression and infant mortality [5].

Previous literature has focused how postpartum behaviors, e.g., supplementing infants’ diets instead of exclusive breastfeeding, attribute to the risk of having an ill infant with diarrheal, febrile or other infectious illnesses, which are the leading causes of under-five mortality in Kenya [4, 8, 35, 37, 38]. However, our study focused on the relationship between antenatal depression and infant morbidity, and we found antenatal depression correlated with a higher risk of having an ill infant necessitating a clinic or hospital visit. One study conducted in Pakistan found an increased risk of ≥5 diarrheal episodes per year for infants whose mothers had antenatal depression [39]. Yet, this study emphasized that a unidirectional relationship between maternal mental health and infant well-being cannot be assumed since poor infant health may act as a maintaining factor for a mother’s depressive symptoms [39]. A more recent longitudinal study in Ghana and Cote d’Ivoire demonstrated a 32% higher risk of febrile illness (defined as temperature ≥ 37.5 °C and an unequivocal medical diagnosis severe enough to warrant a prescription from the attending pediatrician) amongst infants of women with perinatal depression as compared to infants of women without depression [40]. This study was unique in that it had one antenatal and two postpartum assessments of depression, and the most recent antecedent depressive episode was used to assess the relationship between maternal depression and febrile illness in infants [40]. Thus, our study supports these previous findings that in utero exposure to maternal antenatal depression, as well as ongoing depression after birth, may have lasting effects on infant health. Physiologic changes related to antenatal depression, i.e., higher levels of maternal cortisol, alterations in the maternal hypopituitary axis and catecholamine function, could predispose infants to developing less robust immune systems and may lead to higher rates of illness [5, 36]. Further work investigating the relationship between depressive symptoms preceding delivery and infant outcomes is necessary to further understand how infants and their health trajectories are impacted by mothers with antenatal depression.

Typically, depression is characterized by social withdrawal and isolation, leading to reduced communication and weakened interpersonal relationships. As a group, mothers with antenatal depression and those with persistent perinatal depression sent significantly less total and less long SMS messages, respectively, compared to their counterparts without depression. Moving forward, tracking messaging behaviors could allow for the development of a mechanism to screen for depressive symptoms amongst women with a lower SMS response rate. Another feature of an mHealth system could provide more tailored messages to women with depressive symptoms to provide better individual support and psychoeducational messages. We currently have integrated this approach by incorporating a new depression track into a large, randomized trial of Mobile WACh NEO. Women with antenatal and postpartum depression now receive specific tracks of messaging that address their mental health condition longitudinally and attempt to engage women around these symptoms. Further qualitive analysis of the message content would enhance our understanding of how mothers with depressive symptoms communicate via this platform as well as allow for the incorporation of diagnostic tools and therapeutic modalities. Additionally, eliciting more feedback about women’s experiences with the messaging platform to identify barriers to use, likes and dislikes of this communication modality, or other general reflections about interactive SMS messaging would provide valuable information about utility and customization of such mHealth interventions in the future.

Women with persistent perinatal depression had a non-significant trend for fewer nurse consultations than women with antenatal or no depression. All women who initiated a nurse consult for any reason found them helpful. There was little difference in the topics for which women with or without depression consulted the study nurse, except women with antenatal depression were less likely to consult the nurse about infant health questions. A similar trend was found amongst mothers whose infants were seen at a clinic/hospital for an illness or whose infants were admitted after delivery. In this group of women with ill infants, mothers with antenatal depression were significantly less likely to have consulted the nurse about their infant’s health. This raises questions about the ways in which antenatal depression affects prenatal and infant care engagement and overall treatment-seeking behavior. Further investigation into health-seeking behaviors of women with perinatal depression, especially antenatal depression, is warranted, and there should be a continued effort to explore how mHealth interventions can benefit prenatal and postnatal engagement of women regardless of their depression status.

## Limitations

Our findings should be interpreted in the context of our study’s limitations. One limitation is selection bias. There were 1039 eligible women and 800 women enrolled in the study. Those who were eligible but did not enroll mainly noted they did not feel they had sufficient time to participate in the study or were otherwise busy. One-hundred forty-three women (18%) were lost to follow-up before their exit visit, and 83 women (10%) attended their exit visit by phone and did not complete an EPDS survey because phone-based collection of EPDS data was not approved. A total of 226 women (28%) were therefore missing postpartum EPDS data. It is important to note that 64 of the 143 women lost to follow-up had antenatal depression and another 29 (out of 83) women who had some follow-up data but did not complete a second EPDS also had antenatal depression (see Supplementary Table A in Appendix). Thus, 93 (41.2%) of the 226 women who do not have follow-up information had antenatal depression as compared to the 163 (28.5%) of women in the retained cohort who had antenatal depression (see Supplementary Table B in Appendix). This level of missingness and difference between those with vs. without follow-up data means our findings on postpartum depression are limited by selection. Additionally, sample sizes for some outcomes were small, i.e., persistent perinatal depression, WLWH and infant mortality, making it difficult to draw robust conclusions about these groups/outcomes.

It is important to note that we did not conduct diagnostic evaluation to identify depression; we used the Edinburgh Postnatal Depression Scale (EPDS) to screen for depressive symptoms. Only two EPDS scores were collected for each participant, at baseline and at the end of the study at 14 weeks. Follow up time after delivery was limited to one timepoint of data collection, measuring only immediate maternal and infant outcomes. The Diagnostic and Statistical Manual of Mental Disorders, Fifth Edition (DSM-5) only allows for the diagnosis of a major depression with peripartum onset when onset of symptoms occurs during pregnancy or in the 4 weeks following delivery [41]. Our second EPDS score was not collected until 14 weeks postpartum, outside of the DSM-5 timeframe for diagnosis. Thus, our assertion of perinatal depression in this study relies on a screening tool, which is suggestive of an underlying pathology of depression but not a definitive diagnosis as outlined by the DSM-5 criteria. However, the EPDS has been validated for use in Kenya [42] and literature suggests that the presence of depressive symptoms negatively influences maternal and child health outcomes in a manner similar to clinical depression [4].

Finally, the majority of this analysis relies on self-reported answers to questions. Self-reported data is subject to bias (i.e., reporting bias and social desirability bias), which may result in exaggerated and/or understated answers. Depression itself may contribute to memory bias [43]. Subsequently, SMS message data and self-reported data are not comparable but should reflect similar trends.

## Conclusions

This study sought to characterize perinatal depression by determining its risk factors and impact in a longitudinal cohort of Kenyan women who participated in a two-way mobile communication system with a trained nurse. Our results are consistent with previous research, that abuse during pregnancy, lower educational achievement, and undesired pregnancy are associated with a higher risk of perinatal depression. We did not find associations between perinatal depression and WLWH, adolescents, and non-married/non-cohabitating women. Women with antenatal depression were more likely to have ill infants but equally as likely to have a preterm birth. Overall, women with antenatal depression and persistent perinatal depression sent less total SMS messages and less long SMS messages. All participants felt advice from the study nurse was helpful. Future studies should incorporate screening, diagnosis, and treatment of perinatal depression at multiple time points into standard care and investigate how depression impacts neonatal health in the immediate postnatal period. To advance perinatal depression work conducted here and in previous studies, we recommend that more longitudinal perinatal depression studies explore how to support and engage with expectant mothers through various modalities, including but not limited to mHealth interventions.

## Supplementary Information

**Additional file 1: Supplementary Table A.** Baseline Characteristics for Total and Retained Cohorts. Participant baseline characteristics for the 798 participants with complete enrollment data and for the 572 participants with two completed EPDS surveys. **Supplementary Table B.** Baseline Characteristics of Excluded vs. Retained Cohorts. Comparison of participant baseline characteristics between the 226 participants who were excluded because they did complete a second EPDS survey, and thus did not have a postpartum depression outcome, and the 572 participants included in the study who had two completed EPDS surveys.

## Data Availability

The datasets used and analyzed in this study are available from the corresponding author on reasonable request.

## Abbreviations

AAS: Abuse Assessment Screen
ANC: Antenatal care
aRR: Adjusted relative risk
C-section: Caesarean section
CI: Confidence interval
DD: Delivery date
EDD: Estimated delivery date
EPDS: Edinburgh Postnatal Depression Scale
GEE: Generalized estimating equation
KSh: Kenyan shilling
LMICs: Low- and middle-income countries
MDD: major depressive disorder
mHealth: Mobile health
Mobile WACh NEO: Mobile Solutions for Women, Adolescents and Children’s health: Neonate
RR: Relative risk
SMS: Short message service
USD: United States Dollar
WHO: World Health Organization
WLWH: Women living with HIV
