# Supplementary material for: Perinatal depression and its impact on infant outcomes and maternal-nurse SMS communication in a cohort of Kenyan women
Source: BMC Pregnancy Childbirth. 2022 Sep 22;22:723. doi: 10.1186/s12884-022-05039-6 (PMC9494796; doi:10.1186/s12884-022-05039-6)
Supplement: Supplementary file 1 — Additional file 1: Supplementary Table A. Baseline Characteristics for Total and Retained Cohorts. Participant baseline characteristics for the 798 participants with complete enrollment data and for the 572 participants with two completed EPDS surveys. Supplementary Table B. Baseline Characteristics of Excluded vs. Retained Cohorts. Comparison of participant baseline characteristics between the 226 participants who were excluded because they did complete a second EPDS survey, and thus did not have a postpartum depression outcome, and the 572 participants included in the study who had two completed EPDS surveys. [file 12884_2022_5039_MOESM1_ESM.pdf]

APPENDIX:

Supplementary Table A. Baseline Characteristics for Total and Retained Cohorts

Variables	Total Cohort (n = 798)<br>*n* (%) or median (IQR)	Retained Cohort (n = 572)<br>*n* (%) or median (IQR)
**Age in years**	24 (21- 29)	25 (22-29)
**Adolescents**	97 (12.3%)	58 (10.2%)
**Marital Status**<br>Not Married/Cohabitating	101 (12.9%)	64 (11.3%)
**Education level**<br>Less than primary education	81 (10.2%)	47 (8.2%)
**Employment Status**<br>Unemployed	563 (70.6%)	394 (68.9%)
**Primigravida**	294 (36.8%)	194 (33.9%)
**Number of Living Children**	1 (0-2)	1 (0-2)
**History of Miscarriage**	83 (10.4%)	65 (11.4%)
**Undesired Pregnancy**	281 (35.3%)	195 (34.2%)
**Never Used Family Planning**	263 (33.0%)	160 (28.0%)
**Median monthly household income in KSh**	10,000 (4625-14,000)	10,000 (6000-14,000)
**Experiencing abuse during pregnancy**	30 (3.8%)	19 (3.3%)
**Living with HIV**	52 (6.6%)	36 (6.3%)
**Distance from home to clinic ≥30 min**	541 (67.9%)	358 (62.7%)
**Share the phone with someone**	141 (17.7%)	81 (14.2%)
**Antenatal depression (EPDS ≥ 10 at baseline)**	256 (32.1%)	163 (28.5%)

Supplementary Table B. Baseline Characteristics of Excluded vs. Retained Cohorts

Variables	Excluded Cohort (n = 226)<br>*n* (%) or median (IQR)	Retained Cohort (n = 572)<br>*n* (%) or median (IQR)	p-value
**Age in years**	23 (20-27)	25 (22-29)	<0.0001*
**Adolescents**	39 (17.5%)	58 (10.2%)	0.0074*
**Marital Status**<br>Not Married/Cohabitating	37 (16.9%)	64 (11.3%)	0.048*
**Education level**<br>Less than primary education	34 (15.0%)	47 (8.2%)	0.0060*
**Employment Status**<br>Unemployed	169 (75.1%)	394 (68.9%)	0.10
**Primigravida**	100 (44.2%)	194 (33.9%)	0.0082*
**Number of Living Children**	0 (0-1)	1 (0-2)	0.0055*
**History of Miscarriage**	18 (8.0%)	65 (11.4%)	0.20
**Unwanted Pregnancy**	86 (38.2%)	195 (34.2%)	0.33
**Never Used Family Planning**	103 (45.6%)	160 (28.0%)	<0.0001*
**Median monthly household income in KSh**	9000 (1500-14,000)	10,000 (6000-14,000)	0.13
**Experiencing abuse during pregnancy**	11 (4.9%)	19 (3.3%)	0.41
**Living with HIV**	16 (7.1%)	36 (6.3%)	0.82
**Distance from home to clinic ≥30 min**	183 (81.0%)	358 (62.7%)	<0.0001*
**Share the phone with someone**	60 (26.6%)	81 (14.2%)	<0.0001*

*denotes a significant p-value <0.05
